# Medial Gastrocnemius Tenocutaneous Island Flap: A Salvage Option After Free Flap Compromise in the Lower Limb

**DOI:** 10.7759/cureus.70153

**Published:** 2024-09-25

**Authors:** Ee Theng Khoo, Muath Mamdouh Mahmod Al-Chalabi, Siti Fatimah Noor Mat Johar, Wan Azman Wan Sulaiman

**Affiliations:** 1 Reconstructive Sciences Unit, Universiti Sains Malaysia (USM), Kota Bharu, MYS

**Keywords:** exposed bone, island flap, lower limb salvage, medial gastrocnemius flap, tenocutaneous island flap

## Abstract

Lower limb salvage following free flap compromise poses a significant reconstructive challenge, particularly when managing complex defects with exposed bone or hardware. Despite advances in microsurgical techniques, the medial gastrocnemius flap, known for its reliable vascular anatomy and versatility, remains a valuable option in such scenarios. In this case report, we present the effective use of a medial gastrocnemius tenocutaneous island flap for reconstructing a complex defect in the middle third of the leg following partial free flap failure. This approach provided successful coverage of exposed structures, preserved limb function, and prevented further complications. Our experience highlights the adaptability and utility of the medial gastrocnemius tenocutaneous island flap in lower limb salvage, especially for wounds located in the middle third of the leg, underscoring its role as a good option in complex reconstructive cases.

## Introduction

Reconstructing lower limb defects remains a significant challenge due to the limited availability of expendable soft tissue and unpredictable outcomes. Key factors influencing the choice of reconstruction include the nature of the injury, the size of the defect, exposed anatomical structures, limb vascularity, the presence of infection, and potential donor site morbidity. Depending on the complexity and extent of the injury, options for soft tissue coverage range from skin grafting, cross-leg flaps, muscle flaps, and fasciocutaneous flaps to perforator-based flaps and free tissue transfer.

Classical loco-regional muscle flaps continue to be a valuable option for salvage procedures in leg reconstruction, particularly when other alternatives have been exhausted. The gastrocnemius flap, known for its versatility, consistent anatomical characteristics, and capacity to fill dead space, is a key tool in these situations. First described by McCraw et al. in 1978, it is considered the workhorse flap for managing defects in the knee and the proximal third of the leg [1,2]. The versatility of this flap can be further enhanced by dividing the gastrocnemius muscle at its origin or by scoring its fascia [3].

In this case report, we present a complex case of a traumatic middle-third leg defect with exposed bone and hardware, complicated by partial free flap failure. The defect was successfully reconstructed using a medial gastrocnemius tenocutaneous island flap, which allowed for extended flap reach and effective coverage.

## Case presentation

A 33-year-old male patient, a chronic smoker with a 10-pack-year history, sustained an open Gustilo IIIB fracture of the mid-shaft tibia and fibula following a high-impact motor vehicle accident. The anteromedial leg wound measured 15 x 9 cm, with a tibial bone defect of 7 cm. Initial wound management at a district hospital included two cycles of negative pressure wound therapy (NPWT), followed by skin grafting.

One year later, he was referred to our Plastic and Reconstructive Surgery team for left tibial reconstruction. Examination of the left lower limb revealed an apparent limb length discrepancy, with a true length discrepancy of 6 cm. A previous skin graft site, measuring 18 x 8 cm, was noted over the medial aspect of the middle third of the left leg, with discontinuity of the tibial bone. The left tibial reconstruction was attempted using a vascularized osteocutaneous fibula flap. Unfortunately, the patient experienced two consecutive ischemic insults due to hematoma and vasospasm, necessitating flap exploration procedures. Within hours, signs of partial flap ischemia were observed over the proximal portion of the skin paddle. Sutures were promptly released, exposing the underlying orthopedic hardware. Wet Aquacel Ag (ConvaTec, Reading, UK) dressings were applied every three days for two weeks until the demarcation of flap necrosis was achieved. Areas of unhealthy tissues were debrided, resulting in a wound defect measuring 14 x 6 cm with exposed tibia bone and hardware. Medial gastrocnemius myocutaneous flap, with a distally based skin paddle size 11 x 5 cm was harvested and tunneled to cover the wound defect and the underlying exposed structures. The patient remained hospitalized for the next two weeks and was discharged without any complications.

**Figure 1:**
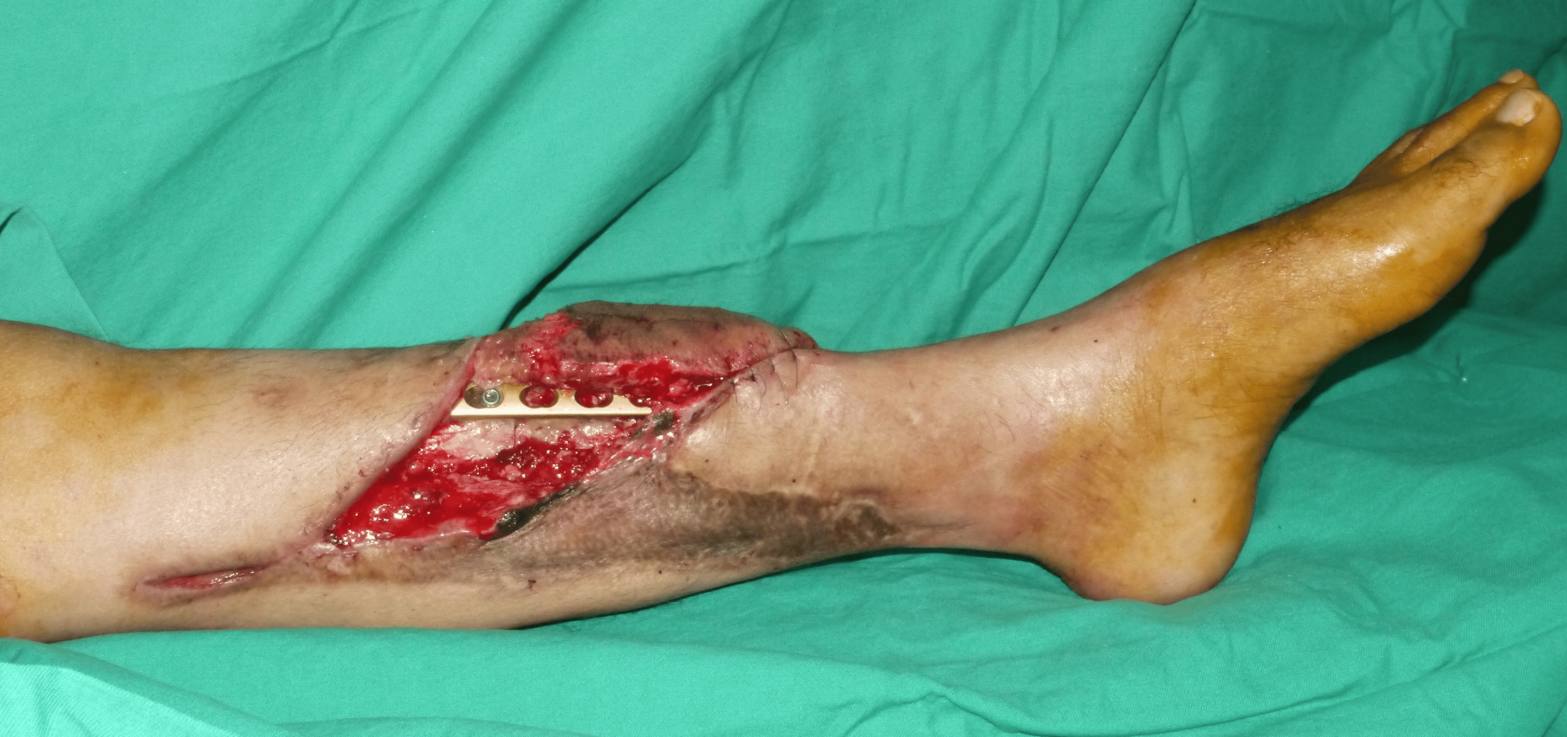
Left anteromedial lower limb wound on the middle third of the leg measuring 14 x 6 cm with exposed tibia bone and hardware.

Wet Aquacel Ag (ConvaTec, UK) dressings were applied every three days for two weeks until the necrotic areas of the flap were fully demarcated. Debridement of the unhealthy tissue was performed, resulting in a wound defect measuring 14 x 6 cm with exposed tibial bone and hardware (Figure 1).

**Figure 2:**
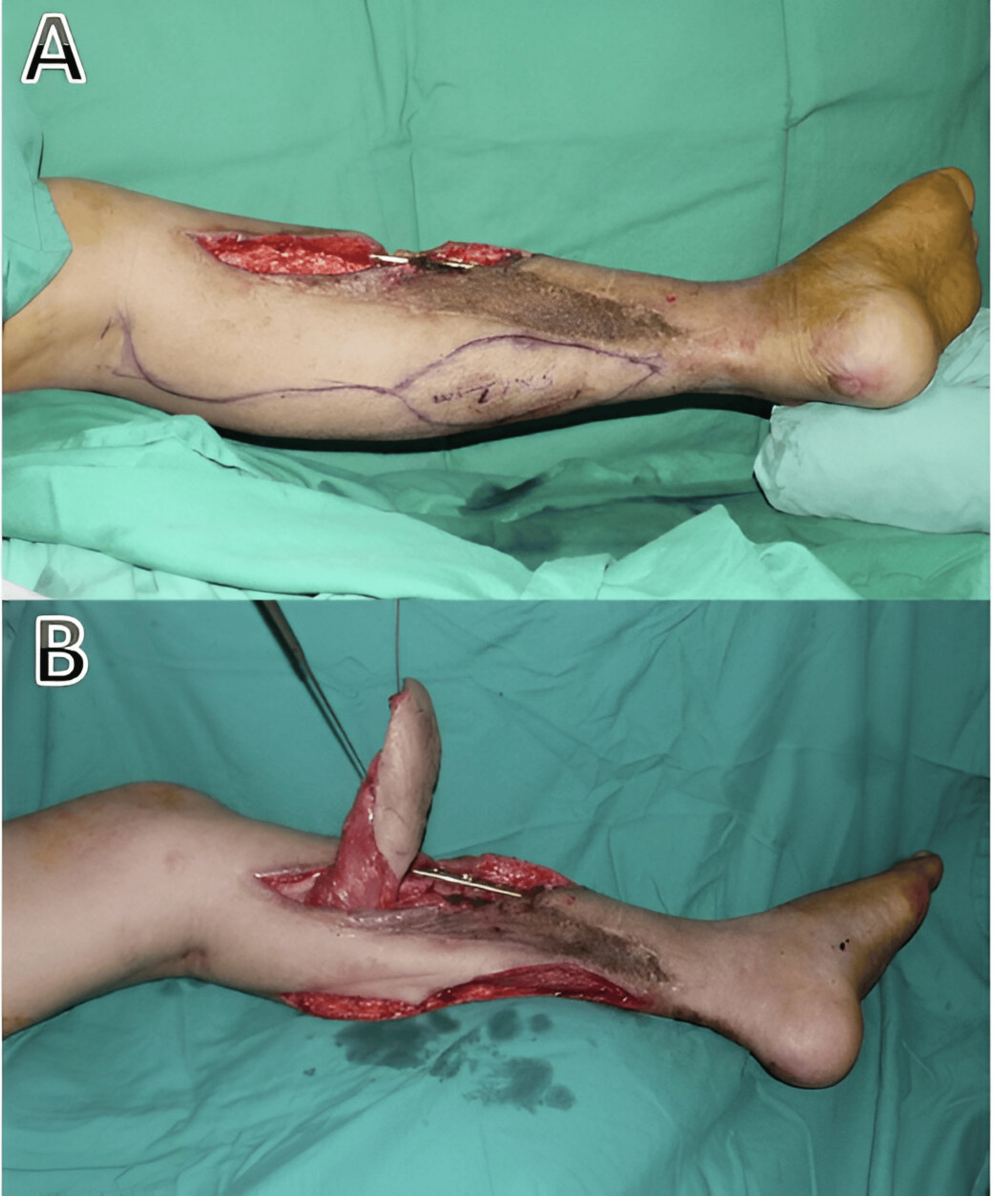
Planning and harvesting of a superiorly based medial gastrocnemius with the island skin attached to the underlying distal tendon.

A medial gastrocnemius muscle flap with tenocutaneous island flap measuring 11 x 5 cm was planned, harvested, and tunneled (Figures 2A, 2B). 

**Figure 3:**
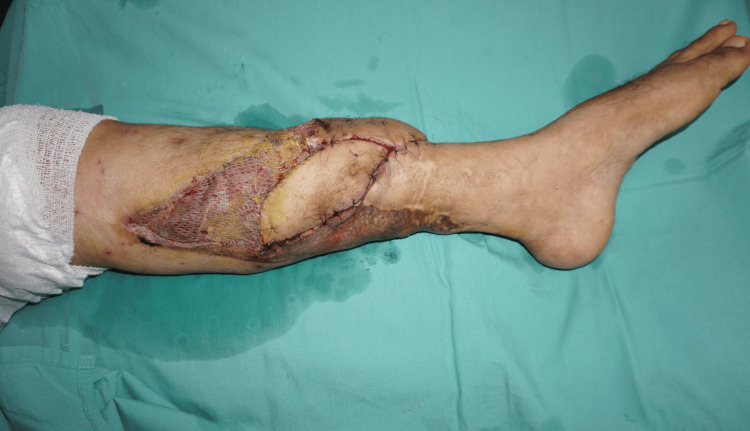
Tunneled flap under the skin bridge covering the defect. A skin graft was applied to the exposed medial gastrocnemius muscle and donor site.

The flap well covered the wound defect and the underlying exposed structures (Figure 3).

**Figure 4:**
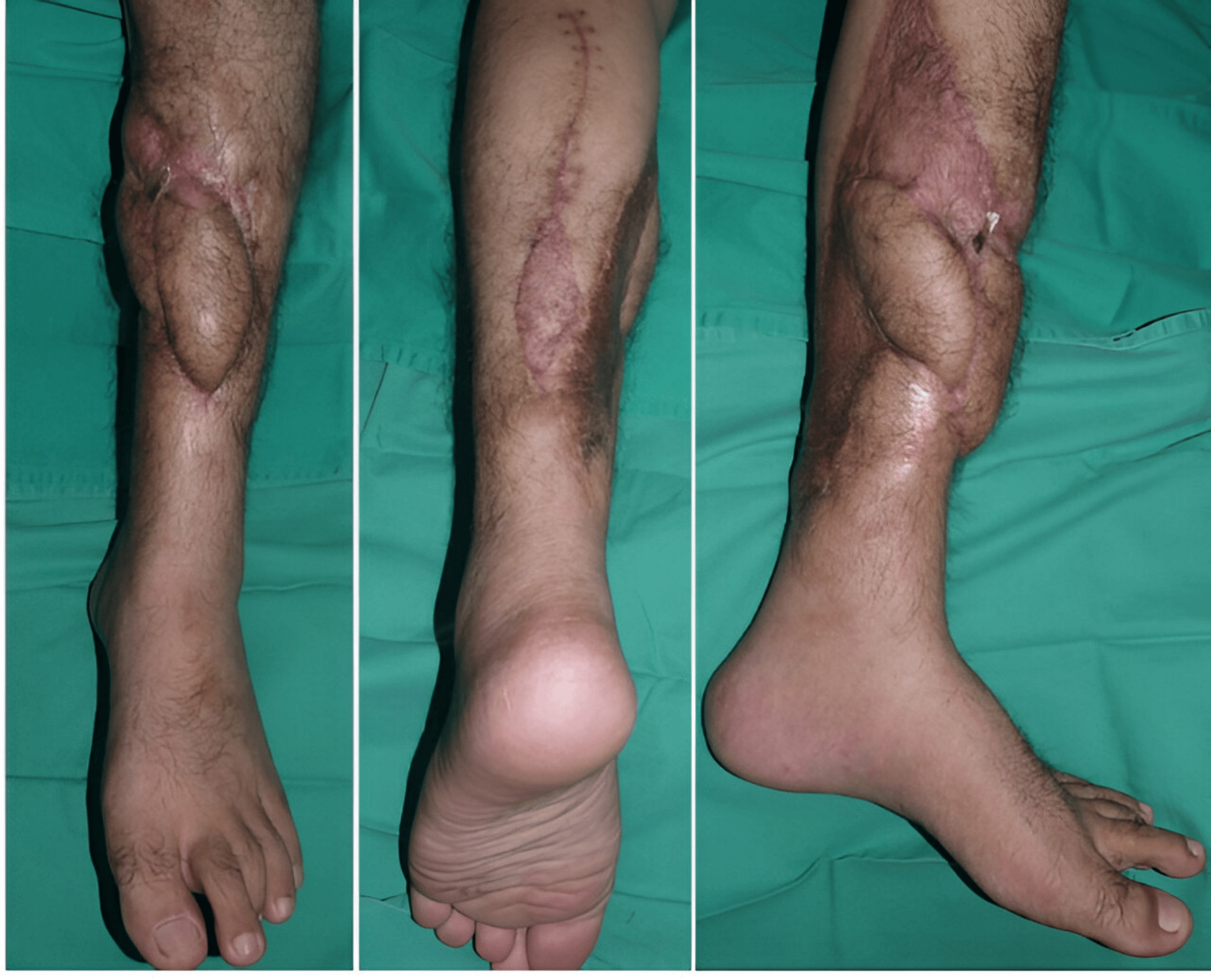
Left lower limb reconstruction at 4 months post-operation shows acceptable and good coverage.

The patient remained hospitalized for two weeks and was subsequently discharged without complications. At the most recent follow-up, the defect and hardware were stable and well-covered (Figure 4).

## Discussion

Reconstructing traumatic lower limb defects is a complex and challenging aspect of reconstructive surgery. These defects often present with insufficient skin coverage, significant soft tissue and muscle loss, exposed vital structures, segmental bone loss, and poor vascularity. Achieving limb preservation, durable soft tissue coverage, stable bone fixation, and early ambulation are essential objectives in devising reconstructive plans [4]. Despite advances in microsurgical techniques, complications such as flap necrosis or failure can still occur, with rates reaching up to 10%, particularly in chronic post-traumatic reconstructions [5,6].

Our case demonstrated the challenges of delayed lower limb reconstruction in an extensive traumatic injury zone, characterized by friable, bleeding-prone wound beds, difficulty in identifying healthy proximal recipient vessels, and refractory vasospasm. These factors ultimately necessitated flap exploration and led to partial flap necrosis, resulting in exposed bone and hardware. While a second free flap is a possible option, it presents greater technical challenges and less favorable outcomes, with failure rates of up to 17%, primarily due to venous thrombosis [6]. Arteriovenous loop construction may be considered for establishing a new recipient site for a free flap; however, this approach carries risks such as vasospasm, vein graft compression, and thrombosis. Additionally, the patient may be exposed to prolonged anesthesia, intensive postoperative monitoring, potential flap re-exploration, and the risk of orthopedic hardware exposure or failure.

The gastrocnemius muscle flap is commonly used as the primary option for reconstructing defects around the knee and upper third of the leg, while the soleus or hemisoleus muscle flap is reliable for covering defects in the middle and lower leg. In certain cases, cross-leg flaps may be considered when other reconstructive options have been exhausted. These flaps provide large, stable soft tissue coverage for extensive leg defects while minimizing additional morbidity to the compromised limb [7]. However, their use is less preferable due to the need for careful patient selection and the associated postoperative complications, including prolonged immobilization, restricted leg movement, joint stiffness, pressure injuries, and deep vein thrombosis.

The pedicled medial gastrocnemius muscle flap remains a valuable and reliable loco-regional option. Its reliability stems from the large muscle belly of the medial gastrocnemius, which contains a higher number of musculocutaneous perforators [1,8,9]. In cases where additional flap length is required, maneuvers such as detaching the medial gastrocnemius muscle at its origin, scoring the muscle fascia, or skeletonizing the vascular pedicle can be employed to enhance reach [3,10]. Unfortunately, most studies focus on its use for reconstructing defects around the knee and upper third of the leg, with limited data available on its application for the middle third leg defects.

To further expand the available options for reconstructing middle third leg defects using the medial gastrocnemius flap, modifications have been reported by Elghamry AH and Chung et al. involving the use of a myotendinous sheath flap and a distal adipo-fascial flap, respectively [11,12]. It has been demonstrated that incorporating up to 8 cm of the Achilles tendon sheath as a vascularized fascial extension can safely enhance the flap's dimensions and provide adequate soft tissue coverage for the middle third of the leg. However, neither technique specifically addresses cases involving exposed hardware or prior complex reconstructions with free tissue transfer. Moreover, these methods are not suitable for our patient due to the need for stable and durable soft tissue coverage over the exposed hardware and the reconstructed free vascularized fibula graft. Both the adipo-fascial and tendinous sheath flaps are relatively thin and require coverage with split-thickness skin grafts. Elghamry AH also reported instances of skin graft loss over the underlying Achilles fascial extension [11]. Inadequate coverage or graft loss poses a risk of infection and hardware failure, potentially leading to increased recipient site morbidity and limited options for further reconstruction.

This case highlights our modification of the medial gastrocnemius flap by incorporating an islanded skin paddle based on the vascularized fascial extension of the Achilles tendon, creating a tenocutaneous island flap. This modification provides an increased arc of rotation, allowing greater reach to the defect while ensuring durable soft tissue coverage over the exposed hardware and vascularized fibula graft. The distal end of the tenocutaneous island flap is positioned 5 cm above the medial malleolus and effectively covers the middle third leg defect. Our patient achieved good functional recovery with no deficits following this technique.

## Conclusions

The medial gastrocnemius tenocutaneous island flap is a reliable option for resurfacing defects in the middle-third of the leg. Our modification broadens the application of this flap, providing durable coverage for large areas while minimizing donor site morbidity. Despite advancements in microsurgery, this technique continues to be a valuable option for complex lower leg reconstruction.
